# Inhibition of IL‐6‐JAK/Stat3 signaling in castration‐resistant prostate cancer cells enhances the NK cell‐mediated cytotoxicity via alteration of PD‐L1/NKG2D ligand levels

**DOI:** 10.1002/1878-0261.12135

**Published:** 2018-01-24

**Authors:** LiJun Xu, XiaoDong Chen, MingJing Shen, Dong‐Rong Yang, Laifu Fang, Guobin Weng, Ying Tsai, Peter C. Keng, Yuhchyau Chen, Soo Ok Lee

**Affiliations:** ^1^ Department of Radiation Oncology School of Medicine and Dentistry University of Rochester NY USA; ^2^ Department of Urology The Second Affiliated Hospital Soochow University Suzhou China; ^3^ Department of Urology Ningbo Urology and Nephrology Hospital China; ^4^ Department of Pathology Ningbo Yin Zhou Hospital China

**Keywords:** castration‐resistant prostate cancer, IL‐6, JAK, NK cell cytotoxicity, NKG2D, programmed death receptor ligand 1, Stat3

## Abstract

To investigate whether IL‐6 signaling affects the susceptibility of castration‐resistant prostate cancer (CRPC) cells to cytotoxic action of natural killer (NK) cells, CRPC cell lines (having different IL‐6 levels) were developed by lentiviral transduction. While observing no secreted IL‐6 level in parental C4‐2 and CWR22Rv1 cells, we found the IL‐6 expression/secretion in these cells was induced after the transduction process and the IL‐6 level difference in C4‐2siIL‐6/sc and CWR22siIL‐6/sc cell CRPC cell sets could be detected. We then found that IL‐6‐knockdown cells were more susceptible to NK cell cytotoxicity than control cells due to lowered programmed death receptor ligand 1 (PD‐L1) and increased NK group 2D (NKG2D) ligand levels. In animal studies, to concur with the *in vitro* results, we found that IL‐6‐expressing cell‐derived tumors were more resistant to NK cell action than the tumors of IL‐6‐knockdown cells. Further, we discovered that JAK‐Stat3 is the most critical IL‐6 downstream signaling that modulates PD‐L1/NKG2D ligand levels in CRPC cells. Furthermore, inhibition of the JAK or Stat3 signaling effectively increased the susceptibility of C4‐2sc and CWRsc cells to NK cell cytotoxicity. We observed the most effective cytotoxicity when the PD‐L1 Ab and JAK inhibitor (or Stat 3 inhibitor) were used together. These results suggest that the strategy of targeting IL‐6 signaling (or its downstream signaling) may enhance the NK cell‐mediated immune action to CRPC tumors, thus yielding clinical implications in developing future immunotherapeutics of exploiting this strategy to treat patients with CRPC.

**Abbreviations**
C4‐2sc: C4‐2 scramble control
C4‐2siIL‐6: C4‐2 IL‐6‐knockdown
CRPC: castration‐resistant prostate cancer
CWRsc: CWR22Rv1 scramble control
CWRsiIL‐6: CWR22Rv1 IL‐6‐knockdown
IVIS: *in vitro* imaging system
LDH: lactate dehydrogenase
MICA: major histocompatibility complex class 1 chain molecule A
NKG2D: NK group 2D
NK: natural killer
PD‐1: programmed death receptor‐1
PD‐L1: programmed death receptor ligand 1
ULBP: UL16 binding protein

## 1 Introduction

Prostate cancer (PCa) is the most commonly diagnosed malignant tumor in men. It often responds to androgen deprivation therapy initially, but progresses from androgen‐dependent PCa to castration‐resistant prostate cancer (CRPC). Although several chemotherapeutic agents have been developed in the treatment for metastatic CRPC (mCRPC), mCRPC mostly remains lethal and refractory to therapy. The development of improved therapeutic approaches for mCRPC is challenging, yet necessary.

While immunotherapy targeting cytotoxic T‐lymphocyte antigen 4 (CTLA‐4) and programmed death receptor 1 (PD1)/PD‐L1 immune check points has shown promising outcomes in the treatment for metastatic melanoma, lung cancer, renal cell carcinoma, and head and neck cancers, clinical trial results for prostate cancer have not been satisfactory (Topalian *et al*., 2012; Yoo *et al*., 2016). A Phase III trial of ipilimumab (CTLA‐4 Ab) in addition to radiotherapy for patients with mCRPC was initiated, but did not show any improvement in overall survival (Kwon *et al*., 2014; Taneja, 2015).

Immunotherapy targeting CTLA‐4 and PD1/PD‐L1 immune checkpoints enhances T cell‐mediated immune reactions against cancer, which appears to be effective for tumors with more mutation burden and mutation‐associated neoantigens (Anagnostou *et al*., 2017; Rizvi *et al*., 2015). The lack of tumor mutation burden and neoantigen were thought to contribute to the lack of CTLA‐4 and PD1/PD‐L1 immune checkpoint‐targeted immunotherapy of PCa. Here, we focused on NK cell‐mediated cytotoxicity in this study as NK cell‐mediated cytotoxicity is independent of tumor mutation burden. NK cells play a pivotal role in exerting antigen‐independent, innate immune responses, which represent the first line of defense against cancer after NK cell infiltration into primary tumors at early stages of tumor development (Abe *et al*., 1998; Kurosawa *et al*., 1995). Indeed, NK cell depletion has led to enhanced tumor formation in mouse models (Smyth *et al*., 2001), supporting the involvement of NK cells in antitumor immunity *in vivo*. Nevertheless, NK cell effects on killing PCa cells are not well understood and reports on NK cell cytotoxicity on PCa are limited. Previously, several attempts have been made to correlate the NK cell activity with the PCa disease state, but no clear conclusions have been drawn (Kastelan *et al*., 1992; Tarle *et al*., 1993). In contrast, Pasero *et al*. (2015) have recently shown that highly effective NK cells are associated with good prognosis in patients with mCRPC.

Studies have shown that overexpression of PD‐L1 may contribute to the poor prognosis of cancer and have linked it to resistance to anticancer therapies (Afreen and Dermime, 2014; Mu *et al*., 2011). High levels of PD‐L1 in tumor cells were reported to be involved in the immune escape process (Iwai *et al*., 2002). In addition to the upregulation of PD‐L1, the downregulation of NKG2D‐activating ligands such as UL16 binding protein 1 (ULBP1), ULBP2, ULBP3, major histocompatibility complex class I chain‐related molecules A and B (MICA and MICB) was reported to be another pathway for tumor cells to escape from NK cell‐mediated cytotoxic actions (Nausch and Cerwenka, 2008). It was also suggested that the downregulated NKG2D in NK cells prevented NK cell recognition by tumor cells (Pasero *et al*., 2016). In addition, Liu *et al*. (2013) showed that activating NKG2D in NK cells mediated antitumor immunity in animal models, and suggested the importance of NKG2D/NKG2D ligand interactions in recognizing tumor cells. Therefore, NKG2D/NKG2D ligand interactions may be an important target in immunotherapy (Spear *et al*., 2013).

The role of IL‐6 in PCa progression has been suggested and may be a candidate for targeted therapy of PCa. IL‐6 was reported to be involved in the regulation of immune reaction and cell growth and differentiation (Culig *et al*., 2005). Activation of signaling pathways of Janus kinase (JAK)/signal transducers and activators of transcription factors (Stats) (Lee *et al*., 2004), mitogen‐activated protein kinase (MAPK) (Culig *et al*., 2005; Godoy‐Tundidor *et al*., 2005), and phosphatidylinositol 3‐kinase (PI3K) (Culig *et al*., 2005; Godoy‐Tundidor *et al*., 2005) by IL‐6 has been reported in various PCa cell lines. IL‐6 is also shown to activate androgen receptor of PCa (Culig *et al*., 2002), even in the absence of androgen (Hobisch *et al*., 1998). In addition, IL‐6 was reported to be involved in the regulation of vascular endothelial growth factor (VEGF) expression (Roberts *et al*., 2013) as well as in the neuroendocrine differentiation process (Xie *et al*., 2004). Moreover, IL‐6 was implicated in epithelial/mesenchymal transition/metastasis of PCa (Gu *et al*., 2014).

Despite the above investigations regarding the association of IL‐6 with prostate cancer, the role of IL‐6 signaling exerting immunosuppressive actions, especially in affecting the NK cell‐mediated cytotoxicity in PCa, has never been studied before. In this study, we explored the role of IL‐6 signaling in promoting resistance to NK cell‐mediated cytotoxicity via modulating PD‐L1 and NKG2D ligand levels in CRPC cells.

## 2 Materials and methods

### 2.1 Cell culture

The CRPC cell lines of C4‐2 and CWR22Rv1 were kindly obtained from Dr YiFen Lee's laboratory at the University of Rochester Medical Center. Cells were cultured in RPMI 1640 containing 10% charcoal‐stripped FBS and maintained in a humidified 5% CO_2_ environment at 37°C. NK92 cell line was purchased from American Type Culture Collection (ATCC, Manassas, VA, USA) and cultured in α‐MEM media containing sodium bicarbonate (M4655; Sigma, St. Louis, MO, USA), IL‐2 (100 units·mL^−1^) (200‐02; Peprotech, Rocky Hill, NJ, USA), inositol (0.2 mm), 2‐mercaptoethanol (0.1 mm), folic acid (0.02 mm), 12.5% horse serum (Sigma), and 12.5% FBS (Hyclone, Logan, UT, USA). For inhibitor studies, JAK inhibitor 1 (5 μm) (CAS457081‐03‐7; Calbiochem, San Diego, CA, USA), Stattic (10 μm) (CAS19983‐44‐9; Calbiochem), LY294002 (5 μm) (440202; Sigma), U0126 (10 μm) (9903; Cell Signaling, Danvers, MA, USA), SB203580 (10 μm) (559387; Sigma) that inhibit JAK, JAK/Stat3, Stat3, PI3K/Akt, MEK/Erk, and MAPK pathways, respectively, were added to the coculture of tumor cells/NK cells. For neutralizing antibody experiments, IL‐6 Ab (6708; Thermo Fisher Scientific, Waltham, MA, USA) or PD‐L1 Ab (329710; BioLegend, San Diego, CA, USA) was added into culture and species‐matched control IgG was used as controls. For testing exogenously added IL‐6 effects, recombinant human IL‐6 (rhIL‐6, 200‐06; Peprotech) was used.

### 2.2 Development of IL‐6‐knockdown and sc control cells by lentiviral transduction

To incorporate IL‐6 siRNA or scramble (sc) control plasmids into C4‐2 and CWR22Rv1 cells, lentivirus construct carrying either sc or IL‐6 siRNA (pLenti‐II vector; Applied Biological Materials Inc., Richmond, BC, Canada) was transfected into 293T cells with a mixture of pLenti‐II‐IL‐6 siRNA, psPAX2 (virus‐packaging plasmid), and pMD_2_G (envelope plasmid) (4 : 3 : 2 ratio) using PolyFect Transfection reagent (Qiagen, Valencia, CA, USA). After C4‐2 and CWR22Rv1 cells were virally infected overnight, the culture media containing the virus were replaced with normal culture media, and maintained under normal cell culture conditions. After subculturing cells, the IL‐6‐knockdown cells were selected by culturing cells in the presence of puromycin (2 μg·mL^−1^) (540411; Millipore, Billerica, MA, USA) and then maintained in media containing 0.1 μg·mL^−1^ puromycin.

### 2.3 Isolation of primary NK cells

Primary NK cells were purified from peripheral blood mononuclear cells (PBMCs) of healthy donors using NK cell isolation kit (130‐092‐657; Miltenyi Biotec, Cambridge, MA, USA) according to the manufacturer's protocol. After isolation, the isolated cells were maintained in IL‐2‐containing NK cell media. The purity of isolated cells (CD56+CD3−) was confirmed with flow cytometric analysis by staining with anti‐CD56‐PE (12‐0267‐41; e‐Bioscience, San Diego, CA, USA) and anti‐CD3‐Cy7 (300429; BioLegend) antibodies.

### 2.4 NK cytotoxicity tests (LDH release‐based)

NK cell cytotoxicity against tumor cells was analyzed using a lactate dehydrogenase (LDH) release assay as described earlier (Shen *et al*., 2017; Yang *et al*., 2017). Cancer cells (2500–5000 cells) were plated, and on the next day, NK cells were added at various ratios (1 : 1, 1 : 5, and 1 : 15, target cells/effector cells) (all sample in triplicate). After 4 h of coculture, an aliquot of 50 μL media was used in LDH cytotoxic assay using the LDH cytotoxic assay kit (88954; Thermo Fisher Scientific). The experimental release was corrected by subtraction of the spontaneous release of effector cells at corresponding dilutions: %cytotoxicity = (experimental value − effector cell spontaneous control − target cell spontaneous control)/(target cell maximum control − target cell spontaneous control) × 100.

### 2.5 NK cytotoxicity tests by colony formation assay

Cancer cells (2500–5000 cells) were plated, and on the next day, NK cells were added at various ratios (1 : 1, 1 : 5, and 1 : 15, target cells/effector cells) (all sample in triplicate). After 4 h of coculture, NK cells were removed and fresh media were added into tumor cells. After 10 days of culture, colonies formed were visualized by crystal violet staining and the colony numbers were counted under microscope.

### 2.6 *In vivo* mouse studies

Orthotopic xenografts were established by orthotopically injecting C4‐2sc (Group 1, *n* = 10) or C4‐2siIL‐6 (Group 2, *n* = 5) cells into castrated nude mice (8 weeks old, male, NCI). For the castration procedure, a sterile surgical scalpel was used for a small incision and the testes were excised for removal. Sterile 3‐0 nonabsorbable sutures were used to close the wounds. Prior to recovery from anesthesia, mice were given buprenorphine at a dose of 0.05–0.1 mg·kg^−1^ subcutaneously for pain. For the orthotopic injection, mice were anesthetized with isoflurane, shaved, and decontaminated with betadine and alcohol, then prepared by a transverse incision made in lower abdomen and the bladder, seminal vesicles, and prostate were partially taken out of the abdominal cavity to expose the anterior prostate. The cancer cells (1 × 10^6^ cells, media/Matrigel, 1 : 1 [v/v], total injection volume 20 μL) were injected into the anterior prostate using a 22‐gauge sterile needle. The exposed urogenital organs were returned to cavity and the incision was closed with surgical clips.

At 7 and 9 days after cancer cell injection, mice were divided into two subgroups (*n* = 5): one subgroup of mice were injected with primary NK cells (5 × 10^6^ per mouse) through tail vein, while the other subgroup of mice were injected with media only. Tumor size was assessed twice a week for 3–4 weeks. Mice were monitored daily for signs of stress indicated by inactivity, slow gait, or lack of grooming. At experimental end points, euthanasia was performed by CO_2_ asphyxiation, consistent with the recommendations of the AVMA Panel on Euthanasia.

At the time of murine sacrifice, tumors of each group were collected and weighed. Tumor tissues were fixed in formalin (10%) for further histologic evaluation, including H&E staining and immunohistochemical (IHC) staining. All animal studies were performed under the supervision and guidelines of the University of Rochester Medical Center's Animal Care and Use Committee in accordance with the NIH guidelines for human care and use of laboratory animals.

### 2.7 Histology and immunohistochemistry (IHC)

Mouse tissues obtained from the animal studies were fixed in 10% (v/v) formaldehyde in PBS, embedded in paraffin, and cut into 5‐μm sections. Human tumor tissues (CRPC stage after prostatectomy) were obtained from the Ningbo Urology and Nephrology Hospital in China and processed similarly. Tumor tissue sections were deparaffinized in xylene solution, rehydrated, and immunostained with the IHC kit (SC2018; Santa Cruz, Santa Cruz, CA, USA). PD‐L1 (MAB1086) and IL‐6 (MAB206) antibodies were obtained from R&D, Batavia, NY, USA. After staining, tissues were counterstained by hematoxylin. Error bars and significance values in IHC staining were obtained by counting positively stained cells in one randomly chosen area of slides of three different stains.

### 2.8 RNA extraction and quantitative real‐time PCR (qPCR) analysis

Total RNA (1 μg) was subjected to reverse transcription using Superscript III transcriptase (Invitrogen, Waltham, MA, USA). qPCR was conducted using the appropriate primers and a Bio‐Rad CFX96 system with SYBR green to determine the mRNA expression levels of genes of interest. Expression levels were normalized to GAPDH level.

### 2.9 Western blot analysis

Cells were lysed in RIPA buffer (50 mm Tris/Cl at pH 7.5, 150 mm NaCl, 1% NP‐40, 0.5% sodium deoxycholate, 1 mm EDTA, 1 μg·mL^−1^ leupeptin, 1 μg·mL^−1^ aprotinin, 0.2 mm PMSF). Proteins (20–40 μg) were separated on 8–10% SDS/PAGE gel and then transferred onto PVDF membranes (Millipore). After the blocking procedure, membranes were incubated with primary antibodies (1 : 1000), HRP‐conjugated secondary antibodies (1 : 5000), and visualized in Imager (Bio‐Rad, Hercules, CA, USA) using ECL system (Thermo Fisher Scientific, 34095). Antibodies used were as follows: p‐PD‐L1 (MAB1086; R&D), JAK1 (pY1022, A7125; Assay Biotech, Sunnyvale, CA, USA), p‐JAK2 (pY1007 + 1008, 601‐670; Abbomax, San Jose, CA, USA), p‐MAPK (9101S; Cell Signaling), p‐Erk (4695; Cell Signaling), p‐MEK (Ser 217/221, 9121; Cell Signaling), p‐Akt (S473, 9271; Cell Signaling), and p‐Stat3 (Y705, ab76315; Abcam, Cambridge, MA, USA), p‐NFκB (S536, ab86299; Abcam), p‐mTOR (09‐345; Millipore), and GAPDH (2118S; Cell Signaling).

### 2.10 Statistics

The data were presented as the mean ± SEM. Differences in mean values between two groups were analyzed by two‐tailed Student's *t*‐test. *P *≤* *0.05 was considered statistically significant.

## 3 Results

### 3.1 Development of C4‐2siIL‐6/sc and CWRsiIL‐6/sc PCa cell lines

**Figure 1:**
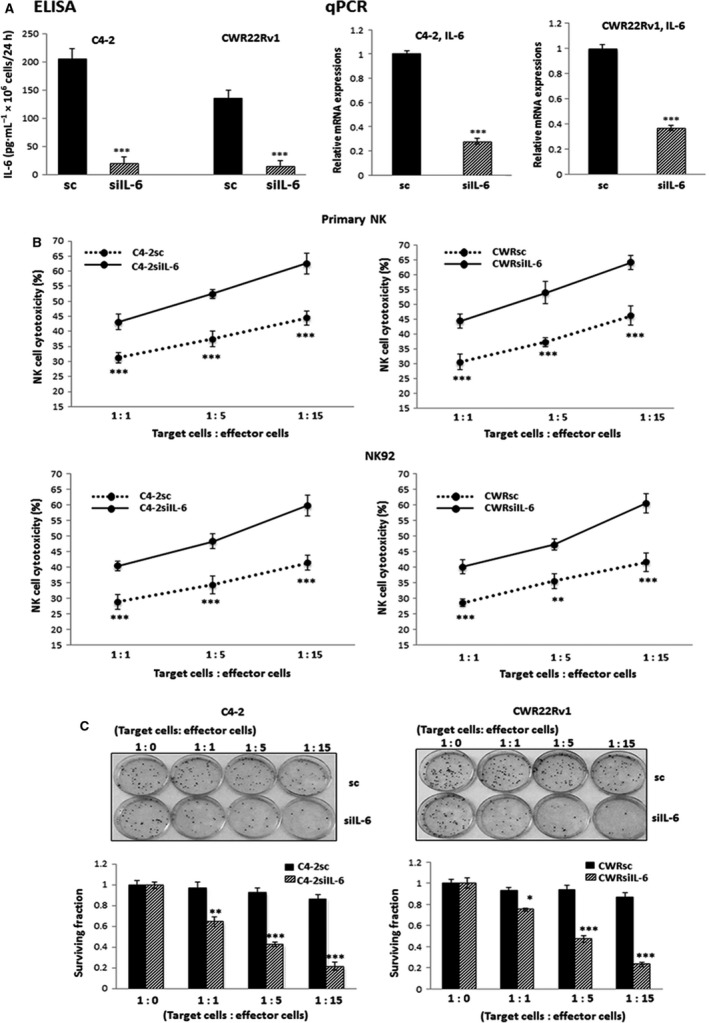
IL‐6 signaling and CRPC cell resistance to NK cell cytotoxicity. (A) IL‐6 expressions after transduction of C4‐2 and CWR22Rv1 PCa cell lines with lentiviral system containing siIL‐6 or sc sequence. IL‐6 expressions (left panel, ELISA result; right panel, mRNA level) in C4‐2siIL‐6/sc and CWRsiIL‐6/sc cell lines were shown. (B) Results of LDH release‐based NK cell cytotoxicity tests to C4‐2siIL‐6/sc and CWRsiIL‐6si/sc cells (upper panel, data with primary NK cells; lower panel, data with NK92 cells). Left panels show data of C4‐2siIL‐6/sc cell set, and right panels show data of CWRsiIL‐6/sc cell set. Average value of triplicate in one experiment was obtained first and the final error bars were calculated from the average values obtained from three independent experiments. (C) NK cell cytotoxicity test by colony formation assay. The survival of C4‐2siIL‐6/sc and CWRsiIL‐6/sc cells after adding various ratios of NK cells was investigated. When colonies become visible, colonies were stained with crystal violet, and colony numbers were counted under the microscope. *P <* *0.05, **P <* *0.01, ***P <* *0.001.

To investigate the implication of IL‐6 signaling in CRPC cells in influencing the susceptibilities to cytotoxic action of NK cells, obtaining CRPC cells lines showing IL‐6 level difference was essential. We found that parental C4‐2 and CWR22Rv1 cells do not secrete IL‐6 (ELISA test, data not shown) although they express high level of mRNA. However, after transduction process using the lentiviral construct carrying siIL‐6 and sc sequence, we observed the induction of IL‐6 secretion and were able to detect significant decreases in IL‐6 levels in siIL‐6‐containing lentivirus‐transduced cells (C4‐2siIL‐6 and CWRsiIL‐6) compared to sc (C4‐2sc and CWRsc) cells (Fig.**** 1, left panel, secreted IL‐6; right panel, mRNA level). These cells were used in further experiments.

### 3.2 IL‐6 signaling plays a role in the resistance to cytotoxic action of NK cells of CRPC cells

NK cell cytotoxicities to C4‐2siIL‐6/sc and CWRsiIL‐6/sc cells were then tested. To monitor NK cell‐mediated cytotoxicity, two different assays, the lactate dehydrogenase (LDH) release‐based NK cytotoxicity test (Decker and Lohmann‐Matthes, 1988; Korzeniewski and Callewaert, 1983; Shi *et al*., 2016; Smith *et al*., 2011) and the colony formation assay (Rong *et al*., 2016), were used. In these experiments, two NK cell sources established NK92 cell line that was known to exhibit high NK cytotoxicity and has been widely used in *in vitro* and in mouse studies (Klingemann *et al*., 2016; Tam *et al*., 1999), and primary NK cells that were isolated from the peripheral blood mononuclear cells (PBMCs) of healthy donors via magnetic beads isolation method, were used. After the isolation of primary NK cells, the purity (higher than 90%) of CD56+CD3− NK cells was confirmed by flow cytometric analyses (data not shown). In LDH release‐based tests, we observed higher resistance of C4‐2sc and CWRsc cells to primary NK cell (Fig. 1B, upper panel)‐ and NK92 cell (Fig. 1B, lower panel)‐mediated cytotoxicities than C4‐2siIL‐6 and CWRsiIL‐6 cells.

Consistent results were obtained in colony formation assays. Higher colony numbers of C4‐2sc and CWRsc cells than those of C4‐2siIL‐6/CWRsiIL‐6 cells were detected after coculture with NK92 cells, suggesting higher resistance of IL‐6‐expressing cancer cells to the cytotoxic action of NK cells than that of IL‐6‐knockdown cells (Fig. 1C, data with primary NK cells).

### 3.3 *In vivo* mouse studies

**Figure 2:**
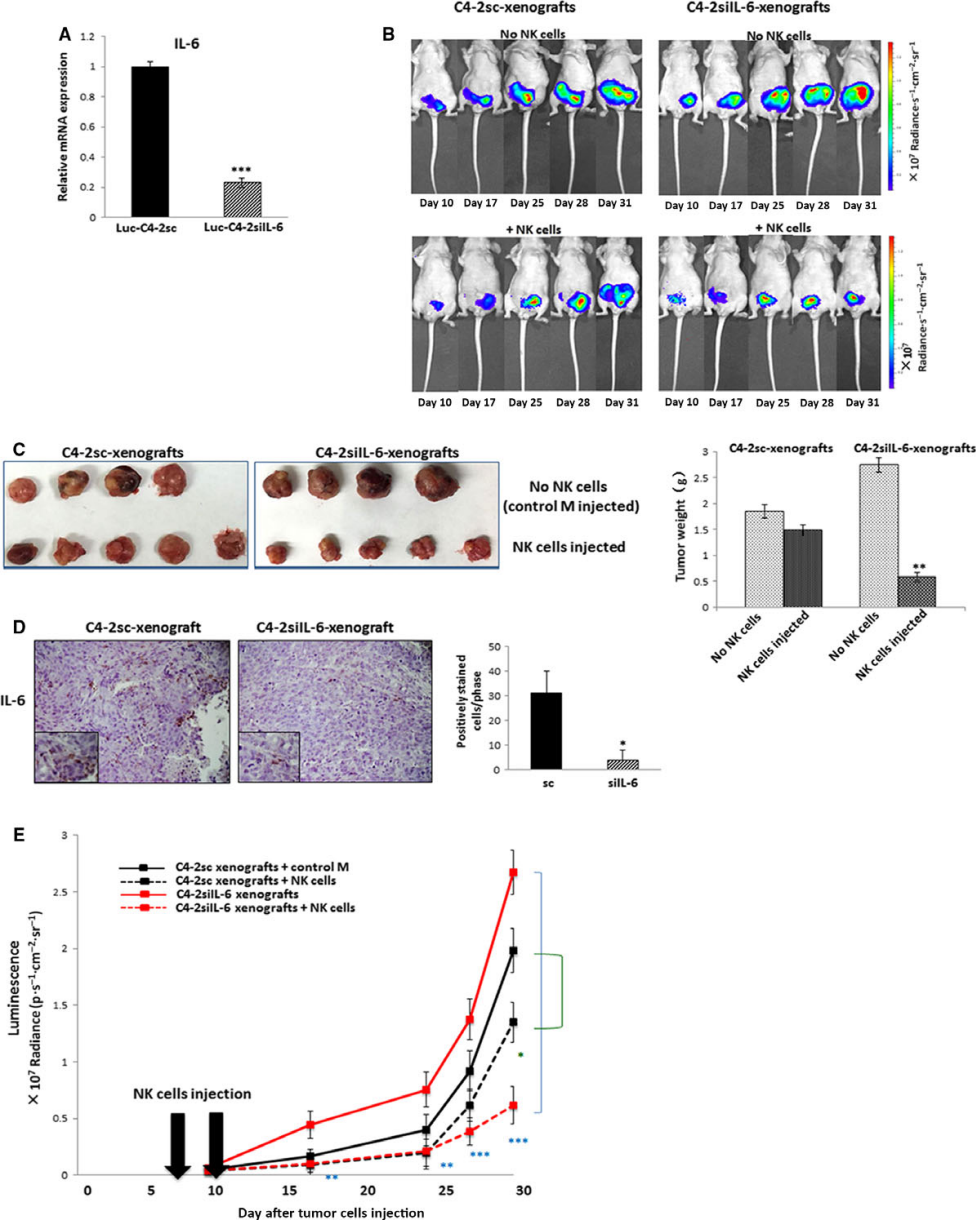
*In vivo* mouse studies showing IL‐6‐mediated resistance of CRPC tumors to NK cell cytotoxic actions. (A) IL‐6 levels in luc‐C4‐2sc and luc‐C4‐2siIL‐6 cells injected into mice. (B) IVIS imaging of representative mice of each subgroup at indicated time points. Upper panel shows imaging of mice of non‐NK cell‐injected group, while lower panel shows imaging of NK cell‐injected mice (left panel, C4‐2sc xenografts; right panel, C4‐2siIL‐6 xenografts). (C) Tumors at sacrifice of mice of each group. Lower panel shows quantitation of the average weight of tumors obtained in mice of each group. (D) IL‐6 IHC staining of tumor tissues. Error bars and significance values were obtained by counting positively stained cells in one randomly chosen phase of slides of three different stains. Magnification, 20× (inlet, 100×). (E) Tumor growth analysis at each time point based on luminescence in IVIS. Luminescence (× 10^7^ radiance·s^−1^·cm^−2^·sr^−1^) was plotted as an indication of tumor growth. *P <* *0.05, **P <* *0.01, ***P <* *0.001.

To confirm *in vitro* results demonstrating the IL‐6 role in rendering the resistance of CRPC cells to NK cell‐mediated cytotoxicity, mouse studies were performed. Luciferase‐tagged C4‐2siIL‐6 and C4‐2sc cells (1 × 10^6^) (*n* = 10) were orthotopically injected into castrated nude mice. Figure 2A shows IL‐6 levels of the luciferase‐tagged C4‐2siIL‐6 and C4‐2sc cells.

Mice were divided into two subgroups (*n* = 5 per subgroup); the test subgroup mice were injected with primary NK cells (i.v.) 7 and 9 days (twice) after tumor cell injection, while the control subgroup mice were injected with control medium. Tumor development was monitored twice a week by *in vivo* imaging system (IVIS) for 3–4 weeks. Figure 2B shows an example of the luminescence of representative mice of each subgroup at indicated time points. We observed significantly smaller tumors in NK cell‐injected mice in C4‐2siIL‐6 cell‐derived xenografts. Such difference was also observed in C4‐2sc cell‐derived xenografts by day 30, but the difference was on a much smaller scale.

Tumors of each subgroup of C4‐2siIL‐6 and C4‐2sc xenografts were obtained at the time of murine sacrifice and tumor sizes were compared. Consistent with luminescence data, we observed significantly smaller tumors in NK cell‐injected siIL‐6 cell‐derived xenografts than in control group mice. A much smaller but significant difference was also found in sc cell‐derived xenografts (Fig. 2C). Figure 2D shows the IL‐6 level in tumors of C4‐2sc and C4‐2siIL‐6 cell‐derived xenografts.

Tumor growths in subgroups of mice were analyzed by plotting luminescence of each time point. We found the growth of C4‐2siIL‐6 cell‐derived tumors significantly reduced in NK cell‐injected mice compared to tumors in the control group, but could not observe significant differences in tumor growth in C4‐2sc cell‐derived tumor growth whether or not NK cells were injected except for the later time point of day 30 (Fig. 2E). All these findings indicate that IL‐6‐expressing tumors are more resistant to the cytotoxic action of NK cells, and confirmed the *in vitro* data suggesting the importance of IL‐6 signaling in determining the susceptibility of tumor cells to cytotoxic actions of NK cells.

### 3.4 IL‐6 upregulates PD‐L1 while simultaneously downregulates NKG2D ligands in CRPC cells

**Figure 3:**
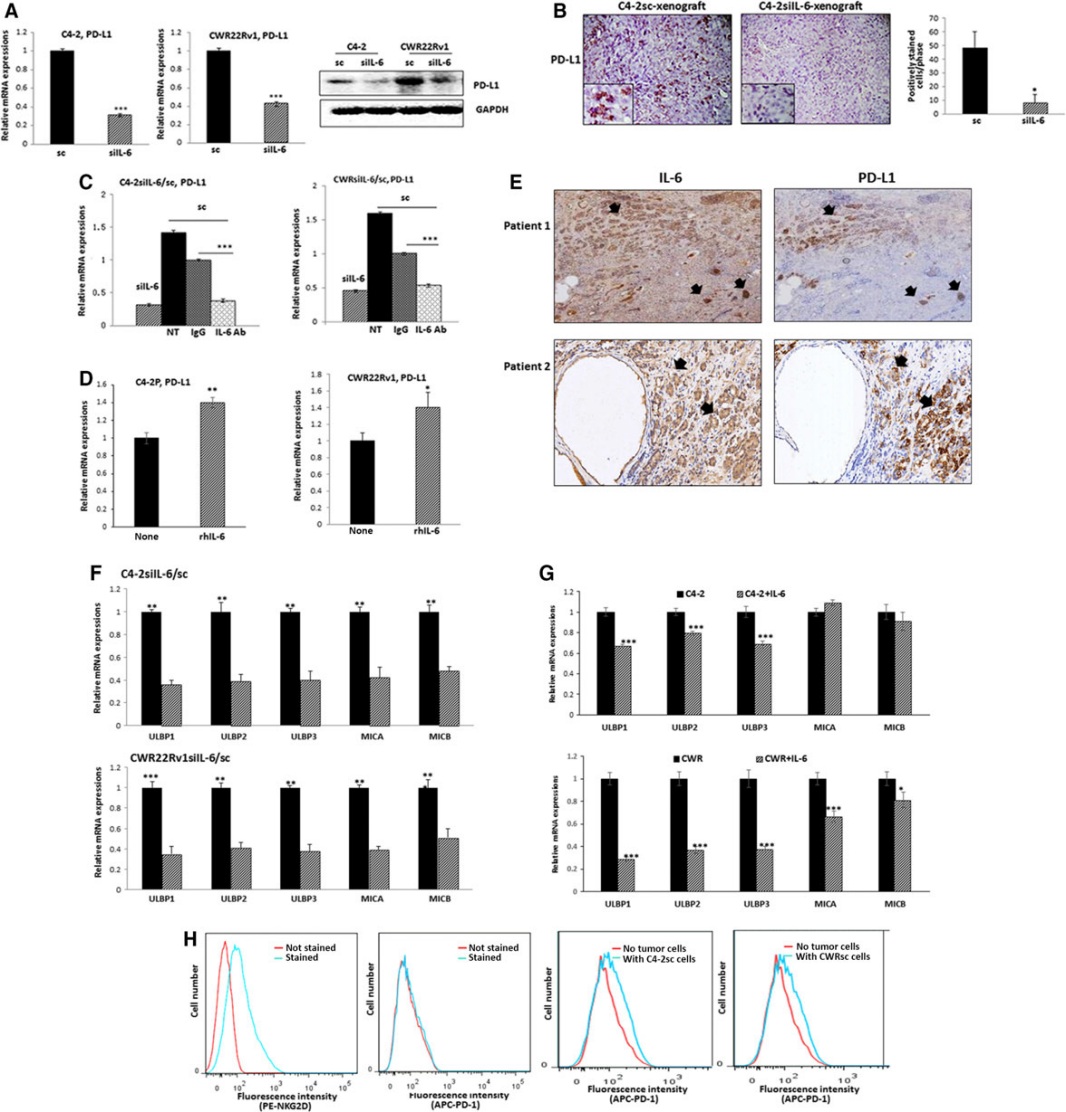
The role of IL‐6 signaling in the upregulation of PD‐L1 and downregulation of NKG2D ligands in CRPC cells. (A) PD‐L1 level in C4‐2siIL‐6/sc and CWRsiIL‐6/sc cell lines (left panel, mRNA level; right panel, protein level). (B) PD‐L1 IHC staining of tumor tissues. Error bars and significance values were obtained by counting positively stained cells in one randomly chosen phase of slides of three different stains. Magnification, 20× (inlet, 100×). (C) Blocking of IL‐6 Ab by neutralizing Ab of IL‐6 and the effect on PD‐L1 level in C4‐2sc and CWRsc cells. Cells were treated with either IL‐6 Ab or control IgG, total RNA extracted, cDNA converted, and the expression of PD‐L1 was compared in qPCR analyses. (D) PD‐L1 level in parental C4‐2 and CWR22Rv1 cells upon the addition of rhIL‐6. Parental cells (C4‐2P and CWR22Rv1P) were treated with rhIL‐6 (20 ng·mL^−1^) and PD‐L1 mRNA level was analyzed. (E) IHC staining of CRPC patient tumor samples. Two sets of adjacent tumor tissues (both samples, CRPC stage, Gleason score 8, patient age 70, Ningbo hospital in China) were stained with IL‐6 and PD‐L1. Arrows indicate the area showing positive staining of two molecules. (F) NKG2D ligand levels in IL‐6‐expressing cells and in IL‐6‐knockdown cells. Levels of five NKG2D ligands in C4‐2siIL‐6/sc and CWRsiIL‐6/sc cells were analyzed in qPCR analyses. (G) NKG2D ligand levels in parental C4‐2 and CWR22Rv1 cells upon the addition of rhIL‐6. Parental cells (C4‐2P and CWR22Rv1P) were treated with rhIL‐6 (20 ng·mL^−1^) and the NKG2D ligand levels (mRNA) were analyzed. (H) Flow cytometric analyses of NKG2D and PD‐1 on NK cells. Left two panels, primary NK cells were stained with PE‐NKG2D or APC‐PD‐1 and positive staining was analyzed. Right two panels, flow cytometric analyses of PD‐1 on NK cells, after coculture with tumor cells (6 h of incubation). Primary NK cells were added into tumor cells (1 : 1 ratio, tumor cells/NK cells) and collected after 6 h of incubation. PD‐1 levels in the collected NK cells were analyzed in flow cytometric analysis (using APC‐PD‐1 Ab). *P <* *0.05, **P <* *0.01, ***P <* *0.001.

The PD‐L1/PD‐1 axis is a well‐known immune checkpoint (Afreen and Dermime, 2014) for T cell‐mediated immunity, and the impact of PD‐L1/PD1 on NK cells remains unclear. We asked whether the IL‐6‐mediated resistance of CRPC cells to NK cell action may be associated with the upregulated PD‐L1 level in these cells. Upon analyzing PD‐L1 levels in IL‐6‐knockdown and control CRPC cells, we detected higher PD‐L1 levels in IL‐6‐expressing C4‐2sc and CWRsc cells than in C4‐2siIL‐6 and CWRsiIL‐6 cells (Fig. 3A, left panel, mRNA level; right panel, protein level). We also detected higher PD‐L1 expression in C4‐2sc cell‐derived than in C4‐2siIL‐6 cell‐derived xenografts (Fig. 3B).

The differences in PD‐L1 levels in C4‐2siIL‐6 vs. C4‐2sc cells and in CWRsiIL‐6 cells vs. CWRsc cells disappeared when C4‐2sc and CWRsc cells were incubated with neutralizing antibody (Ab) of IL‐6 (the species‐matched control IgG as control) to the culture media (Fig. 3C). The IL‐6 implication in the modulation of PD‐L1 level was further confirmed in parental cells. The PD‐L1 levels in C4‐2 and CWR22Rv1 parental cells were increased when recombinant human IL‐6 (rhIL‐6) was added into the culture medium (Fig. 3D).

To further confirm the correlation of IL‐6 with PD‐L1, we examined expression levels of IL‐6 and PD‐L1 in human CRPC tumor tissue samples. We detected high level of IL‐6 expression in areas showing positive staining of PD‐L1 (Fig. 3E), indicating that high IL‐6 level may be responsible for the high PD‐L1 expression in CRPC tumors.

We also investigated a pathway of NKG2D ligand/NKG2D interaction, which has been reported to be important in tumor cell recognition by NK cells (Bae *et al*., 2012) in that lower NKG2D ligand levels played a role in the escape of tumor cells from the NK cell action. When we compared the levels of five NKG2D activating ligands, ULBP1, ULBP2, ULBP3, MICA, and MICB, in IL‐6‐expressing (sc) and IL‐6‐knockdown CRPC cells, we detected reduced levels of these molecules in IL‐6‐expressing sc cells compared with IL‐6‐knockdown cells (Fig. 3F, upper panel, C4‐2siIL‐6/sc cell set data; lower panel, CWRsiIL‐6/sc cell set data). The IL‐6 implication in the modulation of NKG2D ligand level was further confirmed in parental cells. The NKG2D ligand levels in C4‐2 and CWR22Rv1 parental cells were increased when rhIL‐6 was added into the culture medium (Fig. 3G, upper panel, C4‐2 cell data; lower panel, CWR22Rv1 cell data). Such data suggest that the expression of the NKG2D ligands was also modulated by IL‐6 signaling in CRPC cells.

### 3.5 PD‐1 expression in NK cells was induced when incubated with tumor cells

We investigated the PD‐1 level in NK cells as it was reported that PD‐1 is expressed at a high level in T cells (Parry *et al*., 2005), but little is known regarding the PD‐1 level on NK cells. In flow cytometric analyses, we observed positive NKG2D staining in primary NK cells, but could not detect positive PD‐1 staining (Fig. 3H, left panel). We then tested whether PD‐1 expression might be induced when NK cells are exposed to tumor cells. We found that the PD‐1 levels in primary NK cells were increased upon incubation with tumor (C4‐2sc and CWRsc) cells. However, we did not observe significant increase in PD‐1 on NK cells when they were incubated with siIL‐6 cells (data not shown). These findings support our hypothesis that a PD‐L1/PD‐1 interaction exists between IL‐6‐expressing CRPC cells and NK cells.

### 3.6 JAK‐Stat3 signaling is the IL‐6 downstream signaling responsible for the PD‐L1/NKG2D ligand level alteration in CRPC cells

**Figure 4:**
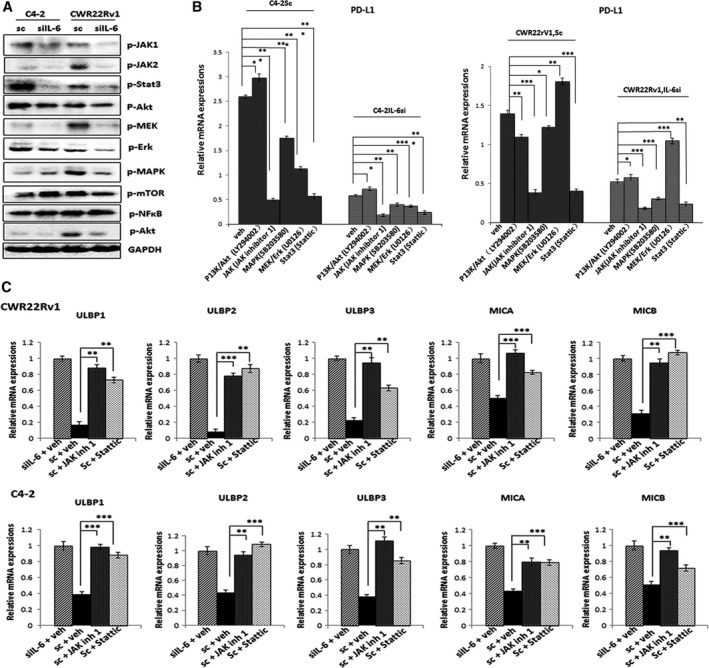
IL‐6 downstream signaling JAK/Stat mediates PD‐L1/NKG2D ligand expressions in CRPC cells. (A) Western blot analyses showing the expression/activation of several IL‐6 downstream signaling molecules in C4‐2siIL‐6/sc and CWRsiIL‐6/sc cells. (B) qPCR analyses in C4‐2siIL‐6/sc and CWRsiL‐6/sc cells with inhibitors of individual candidate signaling pathway. Inhibitors of JAK/Stat (AG490), PI3K/Akt (LY294002), JAK (JAK inhibitor 1), MAPK (SB203580), MEK/Ark (U0126), and Stat3 (Stattic) were used. (C) qPCR analysis of NKG2D ligands in C4‐2siIL‐6/sc and CWRsiIL‐6/sc cells upon treatment with JAK (JAK inhibitor 1) or Stat3 inhibitor (Stattic). Upper panel shows data with C4‐2siIL‐6/sc cell set, and lower panel shows data with CWRsiIL‐6/sc cell set. *P <* *0.05, **P <* *0.01, ***P <* *0.001.

To reveal IL‐6 downstream signaling pathways that are responsible for the IL‐6‐mediated upregulation of PD‐L1/downregulation of NKG2D ligands in CRPC cells, we analyzed the expression/activation of molecules that have been previously reported to be involved in PD‐L1 regulation in C4‐2siIL‐6/sc and CWRsiIL‐6/sc cells. These included JAK1/2 (Bellucci *et al*., 2015; Ikeda *et al*., 2016), Stat3 (Fujita *et al*., 2015; Marzec *et al*., 2008), Akt‐mTOR (Lastwika *et al*., 2016), NFκB (Gowrishankar *et al*., 2015), mitogen‐activated protein kinase/ERK kinase (MEK), extracellular signal‐regulated kinase (ERK) (Yamamoto *et al*., 2009), PI3K/Akt (Xu *et al*., 2014), and MAPK (Noh *et al*., 2015) pathways. We found many signaling pathways, including JAKs, Stat3, MAPK, MEK, Erk, were activated in IL‐6‐expressing cells compared to IL‐6‐knockdown cells (Fig. 4A). Using inhibitors of these signaling pathways, we tested whether blocking of specific signaling could reduce PD‐L1 levels in sc cells. We found that the inhibitor treatment of JAK, MAPK, MEK/Erk, and Stat signaling all reduced PD‐L1 level in sc cells, but the most significant effect was observed with JAK inhibitor 1 (the JAK signaling inhibitor) and Stattic (the Stat3 signaling inhibitor) (Fig. 4B).

We also investigated whether the inhibitors of JAK or Stat3 signaling alter expression of the NKG2D ligands in CRPC cells. We found that the inhibitor treatment increased the NKG2D ligand levels in C4‐2sc and CWRsc cells (Fig. 4C), indicating that the inhibitors of JAK or Stat3 signaling were effective not only in lowering the PD‐L1 levels, but also in increasing the NKG2D ligand levels.

### 3.7 Effects of PD‐L1 Ab or the inhibitors of JAK/Stat3 signaling on NK cell cytotoxicity to CRPC cells

**Figure 5:**
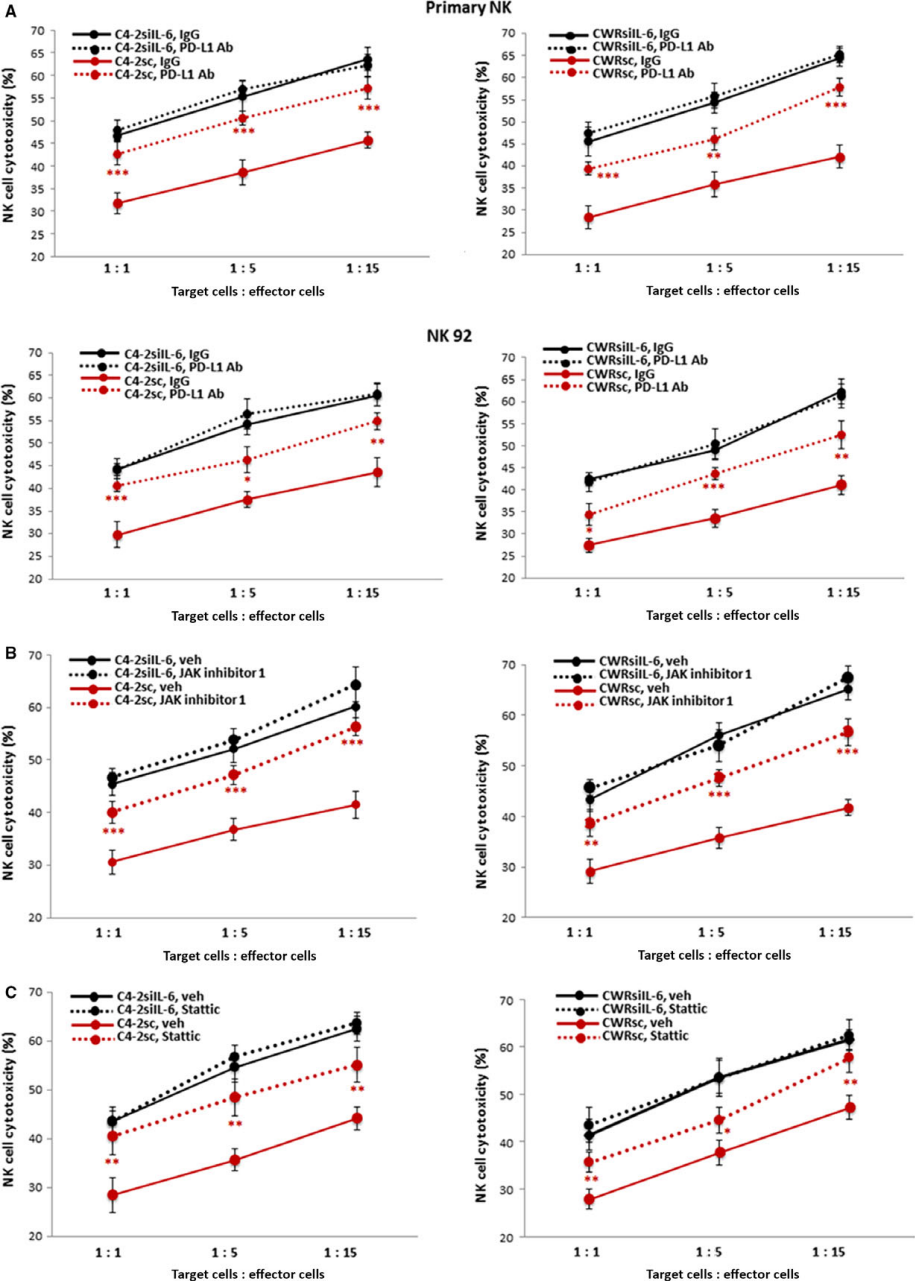
NK cell cytotoxicity to C4‐2sc and CWRsc cells with (A) PD‐L1 Ab, (B) JAK inhibitor, or (C) Stat 3 inhibitor added to cocultures of tumor cells/NK cells. In performing experiments in (A), both primary NK cells (upper panel) and NK92 cells (lower panel) were used. In (B) and (C), primary NK cells were used. Red lines show data with C4‐2sc and CWRsc cells, while black lines show data with C4‐2siIL‐6 and CWRsiIL‐6 cells. Solid lines show data with control IgG or vehicle, while dotted lines show data with PD‐L1 Ab or inhibitors. In all data presentation, C4‐2 cell data are shown in left panels and CWR22Rv1 cell data are shown on right. *P <* *0.05, **P <* *0.01, ***P <* *0.001.

We compared effects of applying PD‐L1 Ab or inhibitors of JAK/Stat3 signaling on NK cell cytotoxicity to C4‐2sc and CWRsc cells. First, the effects of PD‐L1 Ab on NK cell cytotoxicities to C4‐2siIL‐6/sc and CWRsiIL‐6/sc cells were tested. As shown in Fig. 5A (upper panel, with primary NK cells data; lower panel, with NK92 cells data), the addition of the PD‐L1 Ab significantly increased the susceptibility of C4‐2sc and CWRsc cells to NK cells action (red lines), but the susceptibility of C4‐2siIL‐6 and CWRsiIL‐6 cells to NK cell cytotoxicity was not significantly influenced (black lines).

Next, we tested the effects of inhibitors of JAK/Stat3 signaling in increasing NK cell cytotoxicity to C4‐2sc and CWRsc cells. Similar to PD‐L1 Ab effects, we found that both the JAK inhibitor (JAK inhibitor 1) and the Stat3 inhibitor (Stattic) treatment significantly increased the NK cell cytotoxicity to C4‐2sc and CWRsc cells (red lines), but not to C4‐2siIL‐6 and CWRsiIL‐6 cells (black lines). Figure 5B shows the data with JAK inhibitor 1 and Fig. 5C shows the data with Stattic.

### 3.8 Testing effects of combined application of PD‐L1 Ab and inhibitors of JAK or Stat3 signaling in NK cell cytotoxicity to CRPC cells

**Figure 6:**
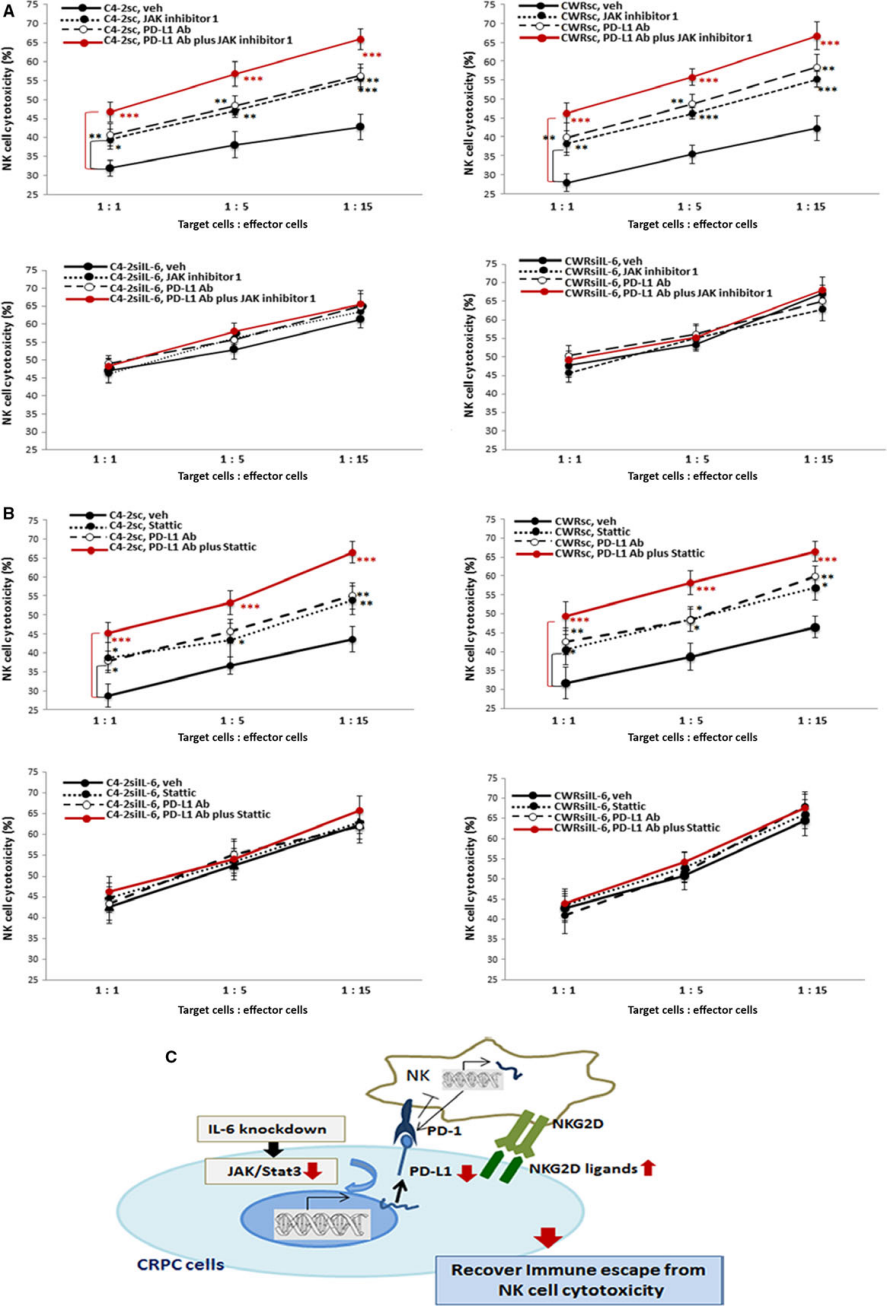
NK cell cytotoxicity to C4‐2sc and CWRsc cells with (A) PD‐L1 Ab and JAK inhibitor or (B) PD‐L1 Ab and Stat 3 inhibitor added together to cocultures of tumor cells/NK cells vs. with PD‐L1 Ab or inhibitor alone. In all experiments, primary NK cells were used as a NK cell source. Upper panels show data with C4‐2sc and CWRsc cells, and lower panel shows data with C4‐2siIL‐6 and CWRsiIL‐6 cells. Red lines show data with combined use of (A) PD‐L1Ab plus JAK inhibitor (JAK inhibitor 1) and (B) PD‐L1 Ab plus Stat 3 inhibitor (Stattic). In all data presentation, C4‐2 cell data are shown in left panels and CWR22Rv1 cell data are shown on right. (C) Summary diagram of the results. *P <* *0.05, **P <* *0.01, ***P <* *0.001.

Finally, we tested whether we could observe enhanced effects when PD‐L1 Ab and the inhibitors of JAK or Stat3 signaling were used together. Figure 6A (upper panels, C4‐2sc/CWRsc cell data; lower panel, parental cell data) shows the NK cell cytotoxicity data of combined use of JAK inhibitor plus PD‐L1, while Fig. 6B shows the data of combined use of Stat3 inhibitor and PD‐L1 Ab (upper panels, C4‐2sc/CWRsc cell data; lower panel, parental cell data). In both cases, we observed that combined use of the JAK or Stat3 inhibitor with the PD‐L1 Ab showed more significant effects than using the PD‐L1 Ab or the JAK/Stat3 inhibitor alone. These results suggest that a strategy of combined application of (a) PD‐L1 Ab and JAK or (b) PD‐L1 Ab and Stat3 inhibitor may have therapeutic potential to target CRPC than the PD‐L1 Ab therapy alone by enhancing the susceptibility to NK cell‐mediated cytotoxicity. Figure 6C is the schematic presentation.

## 4 Discussion

Clinical trials targeting PD‐L1/PD‐1 axis in treating PCa so far have not yielded satisfactory results. A recent clinical trial using PD‐1 Ab (nivolumab) to treat men with mCRPC showed no evidence of improvement while showing clear antitumor efficacy in treating melanoma, renal cell, and non‐small‐cell lung cancer tumors (Topalian *et al*., 2012). The lack of effects of the immunotherapy targeting the PDL1/PD1 axis on T cell‐mediated immunity has been attributed to low tumor mutation burden and low neoantigens (Anagnostou *et al*., 2017; Rizvi *et al*., 2015). While T cell‐mediated immunotherapy is antigen dependent, a strategy to improve NK cell cytotoxicity, which is antigen independent, may be a logical alternative immunotherapeutic approach for the treatment of PCa. To our knowledge, virtually no attempts have been made to improve NK cell cytotoxicity for PCa (Srivastava *et al*., 2008). In our *in vitro* and *in vivo* studies, we have demonstrated a significant NK cell effect in killing PCa cells, supporting our rationale to develop strategies to improve NK cell‐mediated cytotoxicity to tumor cells.

In animal studies, we injected NK cells at early time points, 7 and 10 days after tumor cell injection, which would inhibit early‐stage tumor development. We successfully showed that NK cell action is important in controlling tumor development at an early stage with significantly lower tumor volume in NK cell‐injected mice compared to control (non‐NK cell‐injected) mice. It is also possible that NK cells may affect metastasis (Yang *et al*., 2003); thus, future *in vivo* studies with a focus on testing NK effect on targeting distant metastasis at later stages of PCa will be informative. In addition to the direct cytotoxic effects by NK cells on cancer cells, it was also suggested that early‐appearing tumor‐infiltrating NK cells may play a crucial role in the generation of antitumor T cells (Kurosawa *et al*., 1995). Whether the better tumor control in NK cell‐injected mice we have observed was due to direct NK cell killing of tumor cells or from the activation of T cells to target tumor cells, or the combination of both, should be further investigated in future animal studies.

We used C4‐2siIL‐6/sc and CWRsiIL‐6/sc cell sets in all experiments. Although parental C4‐2 and CWR22Rv1 cells do not secrete IL‐6 (while they express high level of IL‐6 mRNA), these cells secrete IL‐6 after lentiviral infection and showed significant differences in IL‐6 levels (both mRNA and secretion) after transduction process using lentivirus carrying siIL‐6 or sc sequence. IL‐6 expression can be induced as a defensive mechanism against viral infection (Hou *et al*. 2014).

In both *in vitro* and *in vivo* studies, we proved the contribution of IL‐6 signaling in rendering CRPC cell resistance to NK cell cytotoxicity. Although several pathological roles of IL‐6 in PCa progression have been suggested (Culig *et al*., 2002, 2005; Gu *et al*., 2014; Hobisch *et al*., 1998), the role of intratumoral IL‐6 in promoting the resistance to NK cell action has never been shown before. It was interesting to note that we observed more aggressive tumor development and growth in C4‐2siIL‐6 cell‐derived xenografts than in C4‐2sc cell‐derived xenografts. Such observation is consistent with previous reports suggesting an inhibitory effect of IL‐6 in PCa cell growth (Culig and Puhr, 2012; Culig *et al*., 2005), reflecting the complexity of IL‐6 function in cancer cells. Despite the difference in aggressiveness of tumor development, our data clearly demonstrated the IL‐6 role in controlling the susceptibility of CRPC tumors to NK cell cytotoxicity.

In this study, we showed the IL‐6 effect in CRPC cells in modulating the PD‐L1 (upregulation) and NKG2D ligand (downregulation) levels and thus inhibiting the NK cell action. Several reports suggested the influence of cytokines in expanding NK cells and in activating the NK cell action. The cytokines interleukin (IL)‐2, IL‐12, IL‐15, IL‐18, and IL‐21 were used to culture and expand NK cells. Granzin *et al*. (2017) showed that combined treatment of these cytokines affected NK cell maturation, proliferation, survival, distribution of NK cell subpopulations, and its activation. Earlier, Vester *et al*. (1999) showed that IFN‐γ‐inducible protein 10 (IP‐10), monocyte chemoattractant protein 1 (MCP‐1), and lymphotactin were induced upon viral infection and activated NK cell action and Guo *et al*. (2016a) showed that NK cell activity was increased by IL‐12. However, not many reports indicated the cytokine role in developing a resistance to NK cell action.

In this study, we focused on the role of intracellular IL‐6 signaling in developing the resistance of PCa cells to NK cell‐mediated cytotoxicity. It was previously suggested that an immunosuppressive environment impairs NK cell function at multiple levels in PCa (Pasero *et al*., 2016). IL‐6 can be secreted by other types of cells in the tumor microenvironment (TME) including macrophages, endothelial cells, and bone marrow mesenchymal cells. Moreover, we observed the exogenously added IL‐6 effect on modulating the PD‐L1/NKG2D ligand levels in CRPC cells. Therefore, investigating the IL‐6 role secreted by other cell types in TME in the future seems essential.

We observed increased PD‐1 level on NK cells after incubation with CRPC cells. While high PD‐1 levels in T cells and its role in immune evasion was reported (Parry *et al*., 2005; Saito *et al*., 2013), limited information is available regarding PD‐1 expression in NK cells, induction of PD‐1 level in NK cells after viral infection, and induction of PD‐1 after culture in the presence of cytokines (Guo *et al*., 2016b). The discovery of showing the PD‐1 induction in NK cells after incubation with CRPC cells is important as it confirms the existence of high PD‐L1/PD‐1 interaction between C4‐2sc/CWRsc cells and NK cells. This also explains why we observed effects of blocking the PD‐L1/PD‐1 checkpoint on increasing NK cytotoxicity to C4‐2sc and CWRsc cells.

In inhibitor studies, we found that JAK/Stat3 pathway was the most critical IL‐6 downstream signaling pathway that modulated the alteration of PD‐L1/NKG2D ligands in CRPC cells in the same manner, that is, an increase in PD‐L1 and a decrease in NKG2D ligands. We believe identifying the critical IL‐6 downstream signaling pathway is important as the therapeutic approach using anti‐IL‐6 agents may result in complicated and unforeseen untoward outcomes because of the complex role of IL‐6 in the physiological activities on both pro‐ and anti‐inflammatory effects in the immune system (Scheller *et al*., 2011). Thus, we speculate that targeting the JAK/Stat3 signaling pathway may be a better therapeutic option than using the anti‐IL‐6 agents.

Based on our data, applying JAK/stat3 inhibitors, either alone or in combination with anti‐PD‐L1 approach, may be an effective therapeutic approach to improve the efficacy of immunotherapy for prostate cancer. Although PD‐L1 Ab and JAK/Stat3 inhibitors both affect PD‐L1, we speculate that their molecular mechanisms are different; the PD‐L1 Ab is expected to block the PD‐L1/PD‐1 interaction between tumor and NK cells, while the JAK/Stat3 inhibitors will contribute to lowering the PD‐L1 ligands in tumor cells, as we noted that the use of PD‐L1 Ab did not reduce the PD‐L1 level in tumor cells (data not shown). Thus, we believe that through different mechanisms, the application of PD‐L1 Ab and JAK/Stat3 inhibitors together may enhance the therapy efficacy than the use of single agents as we observed in Fig. 6. Targeting the JAK/Stat pathway to treat solid tumors has been previously suggested (Thomas *et al*., 2015). Although our *in vitro* results showed similar effects when JAK or Stat3 pathway was inhibited, we speculate that the combined use of the JAK inhibitor with PD‐1 (or PD‐L1) Ab instead of combining Stat3 inhibitors with PD‐1 (or PD‐L1) Ab to treat patients with CRPC may be a better option for clinical application because of the following reasons: (a) Blocking the JAK pathway is expected to inhibit its downstream Stat 3 signaling by inhibiting the Stat3 Tyr phosphorylation (Hedvat *et al*., 2009), and (b) clinical reports using the JAK inhibitor showed more promising results than the Stat3 inhibitor. Further *in vivo* investigation to prove the effect of this combined therapy in enhancing the NK cell cytotoxicity will be essential to validate the potential of this novel therapy strategy.

Detailed mechanisms that JAK/Stat3 pathway triggers expressions of PD‐L1/NKG2D ligands have not been explored in this study. Ritprajak and Azuma (2015) suggested that JAK/Stat‐PI3K/Akt‐mTOR pathway promotes the transcription of PD‐L1 via activation of transcription factor, S6K. In our studies, we observed that the PD‐L1 expression in CRPC was also affected by inhibitors of Akt and mTOR pathways (although the effect was not as significant as the effects of JAK/Stat3 inhibition), so we speculate that this regulation is possible. It was also suggested that JAK/Stat may directly promote the PD‐L1 transcription via activation of the transcription factor, interferon regulatory factor‐1 (IRF‐1) (Ritprajak and Azuma, 2015). On the other hand, it was also shown that the IL‐6/JAK/STAT3 signaling pathway‐mediated PD‐L1 increase in lung cancer cells was via activation of EGFR‐tyrosine kinase (Zhang *et al*., 2016). This possibility should also be tested.

## 5 Conclusion

In this study, we discovered the IL‐6 role in CRPC cells in rendering the resistance to NK cell‐mediated cytotoxicity due to the alteration of PD‐L1/NKG2D ligand levels. We further found that JAK‐Stat3 is the most critical IL‐6 downstream signaling that is responsible for the modulation of PD‐L1/NKG2D ligands in CRPC cells, so the addition of inhibitors of JAK or Stat3 signaling increased the susceptibility of CRPC cells to NK cell cytotoxicity. The strategy of targeting IL‐6 or its downstream JAK/stat3 signaling, either alone or together with the current PD‐L1 Ab, may be applied to future immunotherapeutics to treat CRPC.

## Author contributions

LX, XC, and MS performed *in vitro* and *in vivo* experiments, statistical analyses, and made the figures. SOL and YT contributed to the generation of knockdown cell lines. LF and GW provided and performed staining of human tissues. D‐RY and PK helped in interpretation of data and reviewed the manuscript. SOL and YC conceived the idea and wrote the manuscript. All authors reviewed and agreed to the information in this manuscript.
